# Activation of Vegetable Oils by Reaction with Maleic Anhydride as a Renewable Source in Chemical Processes: New Experimental and Computational NMR Evidence

**DOI:** 10.3390/molecules27238142

**Published:** 2022-11-23

**Authors:** Francesco Lanero, Bianca Maria Bresolin, Anna Scettri, Marco Nogarole, Elisabetta Schievano, Stefano Mammi, Giacomo Saielli, Alessia Famengo, Alessandra Semenzato, Giovanni Tafuro, Paolo Sgarbossa, Roberta Bertani

**Affiliations:** 1 Dipartimento di Ingegneria Industriale, Università di Padova, Via Marzolo 9, 35131 Padova, Italy; 2 Stazione Sperimentale per L’industria Delle Pelli e Delle Materie Concianti s.r.l., Organismo di Ricerca Nazionale delle CCIAA di Napoli, Pisa e Vicenza, Via Achille Papa 28, 36071 Arzignano, Italy; 3 Dipartimento di Scienze Chimiche, Università di Padova, Via Marzolo 1, 35131 Padova, Italy; 4 CNR—Institute on Membrane Technology, Unit of Padova, Via Marzolo 1, 35131 Padova, Italy; 5 CNR—Institute of Condensed Matter Chemistry and Technologies for Energy, C.so Stati Uniti 4, 35127 Padova, Italy; 6 Department of Pharmaceutical and Pharmacological Sciences, University of Padova, Via Marzolo 5, 35131 Padova, Italy; 7 Unired Srl, Via Niccolò Tommaseo 69, 35131 Padova, Italy; 8 CIRCC—Consorzio Interuniversitario per le Reattività Chimiche e la Catalisi, Unit of Padova, Piazza Umberto I, 70121 Bari, Italy

**Keywords:** vegetable oil, maleic anhydride, Alder reaction, ene reaction, 2D COSY, HSQC, HMBC and 1D CSSF-TOCSY NMR experiments

## Abstract

Vegetable oils are bio−based and sustainable starting materials that can be used to develop chemicals for industrial processes. In this study, the functionalization of three vegetable oils (grape, hemp, and linseed) with maleic anhydride was carried out either by conventional heating or microwave activation to obtain products that, after further reactions, can enhance the water dispersion of oils for industrial applications. To identify the most abundant derivatives formed, trans-3-octene, methyl oleate, and ethyl linoleate were reacted as reference systems. A detailed NMR study, supported by computational evidence, allowed for the identification of the species formed in the reaction of trans-3-octene with maleic anhydride. The signals in the ^1^H NMR spectra of the alkenyl succinic anhydride (ASA) moieties bound to the organic chains were clearly identified. The reactions achieved by conventional heating were carried out for 5 h at 200 °C, resulting in similar or lower amounts of ASA units/g of oil with respect to the reactions performed by microwave activation, which, however, induced a higher viscosity of the samples.

## 1. Introduction

Increasing ecological concerns have highlighted the importance of switching to renewable resources in terms of materials and energy. Vegetable oils, mainly consisting of glycerol esterified with three fatty acids (with saturated and unsaturated C14–C22 carbon atoms), are suitable bio−based and sustainable starting materials for chemical industrial processes because of their availability, biodegradability, inherent safety, sustainability, and affordability [1,2].

Particular attention must be applied to oils containing higher amounts of unsaturated fatty acids [3], i.e., oleic, linoleic, and linolenic chains, which can undergo a wide variety of chemical conversions through the introduction of functional groups such as hydroxyl, epoxide [4,5,6,7,8], or succinic anhydride [9,10,11,12,13,14] moieties. The preparation of flexible epoxy resins has been reported by the reaction of epoxidized linseed oil crosslinked with a mixture of cyclic anhydrides and maleinized oils [15,16,17,18].

Numerous oils can be considered as sources for other chemicals without interfering with the food sector, especially those which are not edible, or those that, because of their poor organoleptic properties, do not have a vast market despite being available in large quantities. Some examples are those obtained as residues in the production of other goods, such as castor [9,12], tung, linseed [11,15], grapeseed [19], and hemp [20,21] oils, even though the latter two can be of interest for their pharmaceutical properties.

After modifications, the activated oils have been used for a wide variety of applications [13,14], such as plasticizers [22,23] or stabilizers for food packaging [11], hydrophobic sizing of paper [24,25,26], epoxy resin-setting agents [15,16,17,18], lubricants, coatings [5], or coupling agents [27].

Furthermore, the functionalization of vegetable oils allows for the aqueous dispersion of non-water-soluble materials to be achieved for different industrial applications, avoiding the need for additional surfactants to stabilize the dispersion, which may interfere with the industrial process [28].

In this context, the reaction of vegetable oils with maleic anhydride (MA) represents an intriguing topic, especially since new, single-step, solvent-free, or catalyzed synthetic routes, not involving benzene, have been developed [29,30] for the synthesis of MA. A unique class of environmentally friendly macromonomers can be formed by the combination of the rich and versatile chemistry of MA and the biodegradable and renewable nature of vegetable oils, forming derivatives bearing the alkenyl succinic anhydride moiety (ASA) bound to the oil chain [31].

It has been recognized that unsaturated fatty acids react with MA upon conventional heating (usually at 200–220 °C for 3–4 h), but both UV [32] and microwave-assisted [33] processes have been investigated. Alternatively, catalytic processes in the presence of Lewis acids have been described [34]. During the reaction, a viscosity increase has been observed, which could be a problem in the industrial scale-up of the process.

The aim of this paper is to compare the products obtained by the reaction of MA with grapeseed (GO), hemp (HO), and linseed (LO) oils carried out with conventional heating or microwave activation to improve the addition of the ASA moiety as much as possible while controlling the polymerization processes in view of the subsequent reaction with NH_2_- or OH-functionalized compounds to prepare new chemically activated materials for industrial applications.

The products were studied through a series of detailed multinuclear and multidimensional nuclear magnetic resonance experiments performed also on methyl oleate, ethyl linoleate, and trans-3-octene as reference compounds. The results were also supported by computational NMR evidence.

## 2. Results and Discussion

### 2.1. Characterization of the Starting Materials

The purity of the reference reactants, trans-3-octene, methyl oleate, and ethyl linoleate was checked by NMR (Figures S1–S3) and determined to be >98%, 85%, and 93%, respectively. In the case of methyl oleate, the lower purity is due to the presence of linoleate as demonstrated by the specific signal of bis-allylic protons.

The composition in terms of the saturated and unsaturated fatty acids of the grapeseed, hemp, and linseed oils was determined by NMR, as shown in the supplementary material (Figures S4–S7, Tables S2 and S3).

### 2.2. The Reaction of Maleic Anhydride with Unsaturated Fatty Acids

**Scheme 1:**
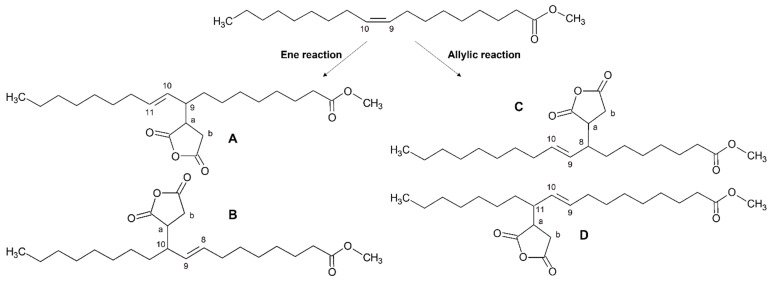
Products of the ene and allylic reactions on methyl oleate.

**Scheme 2:**
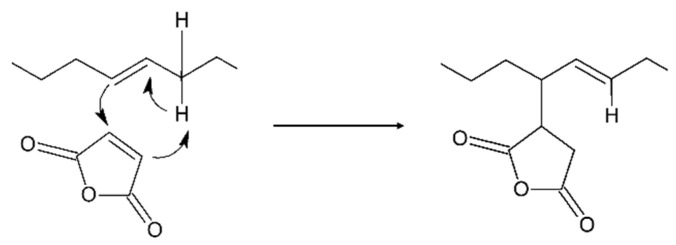
Mechanism of the “ene-Alder” reaction.

The reaction of MA with unsaturated fatty acid moieties in vegetable oils has been reported to proceed through different mechanisms, yielding different products, as summarized in Scheme 1 in the case of the oleic moiety:

(i) The “ene-Alder” reaction which involves the attack of MA (acting as a highly reactive enophile, its double bond being an electron-deficient alkene) on the carbons bearing the double bond (the “ene” moiety) [35,36] thus shifting the double bond on the vicinal carbon and forming a new σ-bond between the two molecules through the general concerted mechanism reported in Scheme 2 [31];

Two possible isomeric structures can be formed: the first bearing the double bond in the C10–C11 position and the alkenyl succinic anhydride (ASA) moiety on C9, the second with the double bond in the C8–C9 position and the ASA moiety on C10. The reaction has been reported to require relatively high temperatures (200–230 °C), involving the transfer of the allylic hydrogen (in C8 or C11) to the enophyle (Scheme 1, ene reaction route), thus forming the species **A** and **B**, respectively.

(ii) A free radical mechanism that involves the attack of MA on the allylic carbons and the maintenance of the double bond in the same C9-C10 position, which can be hydrogenated as well, and bearing the ASA moiety on C8 or C11 [37] (Scheme 1, route allylic reaction), giving rise to the species **C** and **D**, respectively.

**Scheme 3:**
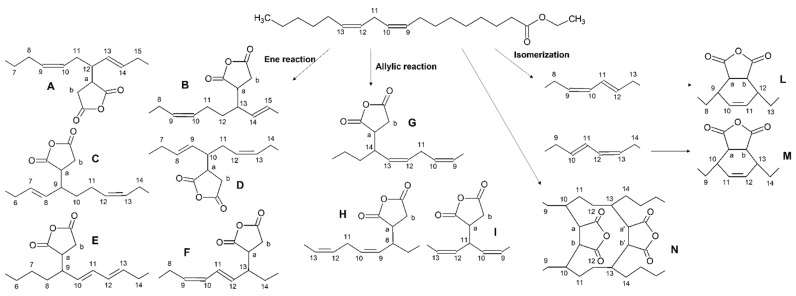
Products of the ene, allylic, and isomerization reactions on ethyl linoleate.

In Scheme 3 additional processes were considered in the case of the linoleic moiety:

(iii) The isomerization of the double bonds to the conjugated systems (Scheme 3, route isomerization) and the subsequent Diels-Alder reaction with the formation of cyclohexene adducts, again in the possible positions C10–C11 or C11–C12, forming the species **L** and **M**, respectively [38,39];
(iv) The attack of MA on C11 [33,40], giving rise to the species **I**;
(v) The formation of ASA inter- or intra-molecular bridges, yielding oligomeric or polymeric species containing the **n** moieties [41].

It has been reported that the ene-reaction is more favorable with respect to the other possible mechanisms, yielding complex product mixtures in any case, including partially or totally hydrogenated species [33].

Recently [42], in agreement with previous observations [43], it was demonstrated that a thermal polymerization of methyl linoleate alone can also occur under anaerobic conditions, probably through a radical mechanism, without the formation of cyclohexene adducts, thus improving the number of species which can be formed during the thermal reactions in the presence [44] or in the absence of MA.

In a recent study, the reactions between soybean or linseed oil with MA were optimized in terms of temperature and time taken to achieve highly functionalized maleated oils in view of a scale-up reaction. It was reported that the reaction carried out in a sealed reactor (without catalyst or solvent, at 200–230 °C for 4 h, under nitrogen, with MA content varying from 1.7 to 4.5 eq per triglyceride) produced dark viscous liquids, while the reactions carried out in an open reactor (at 200 °C for 4 h) produced less viscous and lighter colored final products, even if the MA was almost completely consumed in both cases. Similar results have been achieved on a pilot plant scale. The difference in behavior between sealed and open reactors was explained by a higher oligomerization amount related to the build-up of ethyne in the sealed reactor [40].

### 2.3. Reactions of Maleic Anhydride with Trans-3-Octene

The reaction with MA by the microwave irradiation of trans-3-octene was considered as a model to identify the most abundant products.

**Scheme 4:**
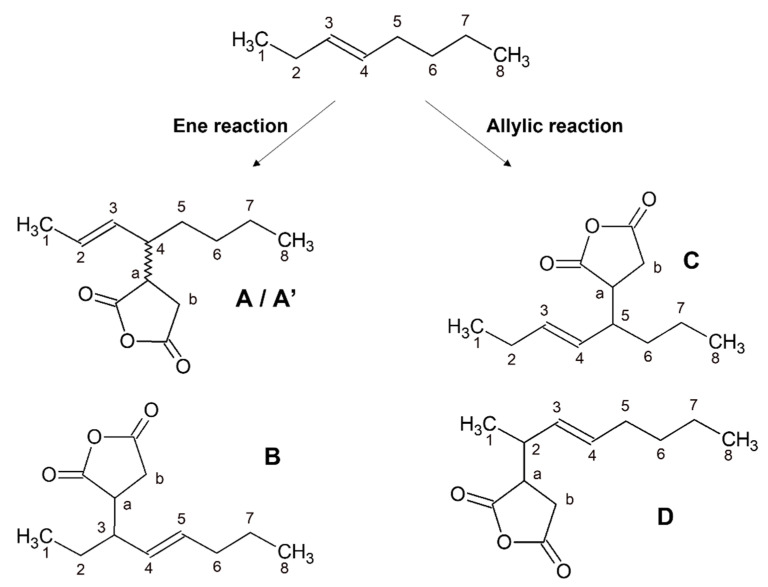
Products of the ene and allylic reactions on trans-3-octene.

**Figure 1:**
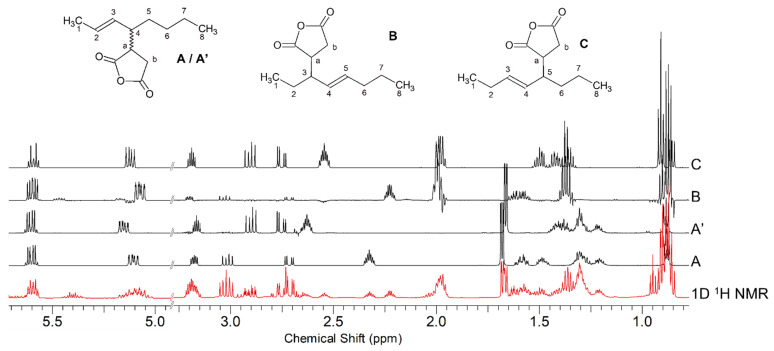
CSSF-TOCSY ^1^H NMR spectra of the reaction mixture of trans-3-octene/MA 1/1.

The compounds that could be formed through “ene” and “allylic” mechanisms from the reaction of trans-3-octene and MA are shown in Scheme 4. Four derivatives were expected, each of them in four stero-isomeric forms, but the only derivatives detected were compound **A** and its diastereomer at C-4, **A’**, and one stereoisomer each for compounds **B** and **C**, while compound **D** was not detected. The accurate chemical shift values of each compound were obtained using CSSF-TOCSY, as demonstrated in Figure 1, and their assignments are reported in Table 1.

To support the assignment of the NMR resonances and the identification of the products obtained by the reaction, we also ran a set of DFT calculations for the carbon and proton chemical shifts of the molecules in Scheme 4. For each compound (**A**, **B**, **C,** and **D**) we built two diastereoisomers, one having the same chirality for the chain and the ASA carbons, and one having the opposite chirality. For all eight molecules, we ran the computational protocol described in the Section 3. Finally, we correlated the calculated vs. experimental values, and from the linear fitting, we obtained the correlation coefficient R^2^ and the Corrected Mean Absolute Error, CMAE, defined as CMAE = Σ_n_|δ_scaled_ − δ_exp_|/n, where δ_scaled_ = (δ_calcd_ − *b*)/*a*. The CMAE measures the distance between the experimental value and the value predicted by the linear fitting, *a* and *b* being the slope and the intercept. This was performed for each calc./expt. data set pair: 8 calculated dat−asets with 4 experimental datasets for a total of 32 correlations.

In Figure S8, we report the R^2^ values of the ^13^C chemical shifts. It appeared that the computational protocol was not able to clearly distinguish between the various compounds: all correlations were similar, probably because the ^13^C experimental data of each molecule were too close to each other with respect to the predictive power of the DFT methods.

**Figure 2:**
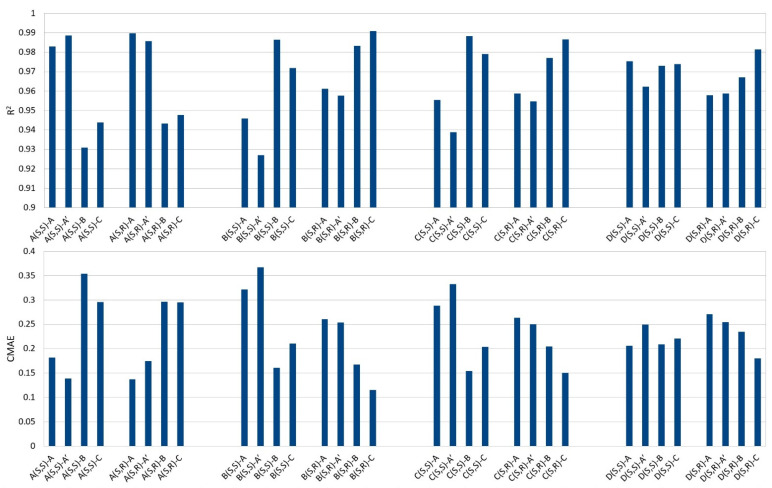
(**Top**) Correlation coefficients, R^2^, obtained from the linear fitting of calculated vs. experimental ^1^H chemical shifts. For each molecule tested, **A**, **B**, **C,** and **D**, we have two diastereoisomers, (S,S) and (S,R), where the first label refers to the chain carbon and the second to the succinic anhydride carbon. Each calculated set is then correlated with the four experimental sets of ^1^H resonances, labeled **A**, **A’**, **B**, and **C** in Table 1. (**Bottom**): Corrected Mean Absolute Errors (CMAE) for the same correlations.

In contrast, the comparison of calculated and experimental proton resonances was more insightful. In Figure 2, we show the correlation coefficients R^2^ for each correlation: the first two sets of experimental values, assigned to the pairs of diastereoisomers **A** and **A’**, show a very high correlation with the calculated values from the two diastereoisomers of **A**. Specifically, both the correlation of the calculated values for the (S,S) isomer with the experimental values labeled **A** in Table 1—and of the calculated values for the (S,R) isomer with the experimental set **A’**—have R^2^ > 0.99. More importantly, the other two sets of experimental data, **B** and **C**, are clearly in disagreement, with R^2^ < 0.95. However, the level of confidence of these computational protocols does not allow us to state with confidence that the experimental set **A** and **A’** corresponds to (S,S) and (S,R), respectively, since the correlation of (S,S) with **A’** and (S,R) with **A** is also high. Similarly, the CMAE values agree with the above conclusions.

On the other hand, the calculations also support the proposal that molecule **D** is not present in the solution: none of the correlations of the calculated values for **D** with the four sets of experimental data is close to 0.99 and none of the CMAE values is below 0.15 ppm.

Less clear-cut results were obtained for molecules **B** and **C** since for both molecules, one diastereoisomer has a high correlation and a low CMAE value with the experimental datasets for both **B** and **C**. Therefore, we cannot distinguish between B and C based on the results of the calculations, so we must rely on the experimental evidence of the double bond position. Overall, the DFT results support the proposed assignment of the four experimental data sets as reported in Table 1.

### 2.4. Reaction of Maleic Anhydride with Methyl Oleate or Ethyl Linoleate

The reactions of MA with methyl oleate and ethyl linoleate in an equimolar ratio were investigated next. The reaction progress was assessed by collecting samples at different times (typically every hour for the conventionally heated samples and every 15 min for microwave-assisted processes), and FTIR spectra were recorded to evaluate the extent of the maleinization reaction. Maleic anhydride is characterized by a sharp, strong absorption at 697 cm^−1^, which decreases during the reactions, while the signals at 1783 and 1857 cm^−1^ are partially covered by the strong absorptions of the ester moieties in the oil. The conversion of MA to ASA can be easily detected through the appearance of a sharp signal at 917 cm^−1^ and a shoulder that becomes a well-defined peak at 1862 cm^−1^ as the reaction proceeds [45].

**Figure 3:**
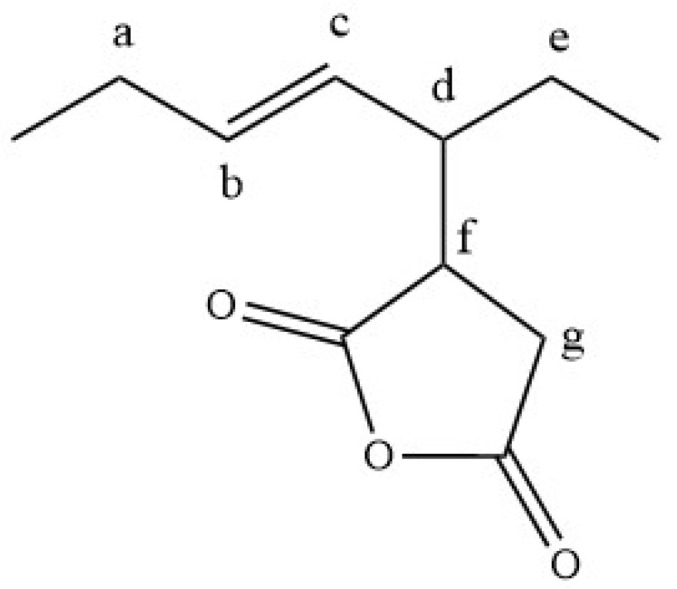
Base structure of the ASA moiety of the oleate’s main product.

**Figure 4:**
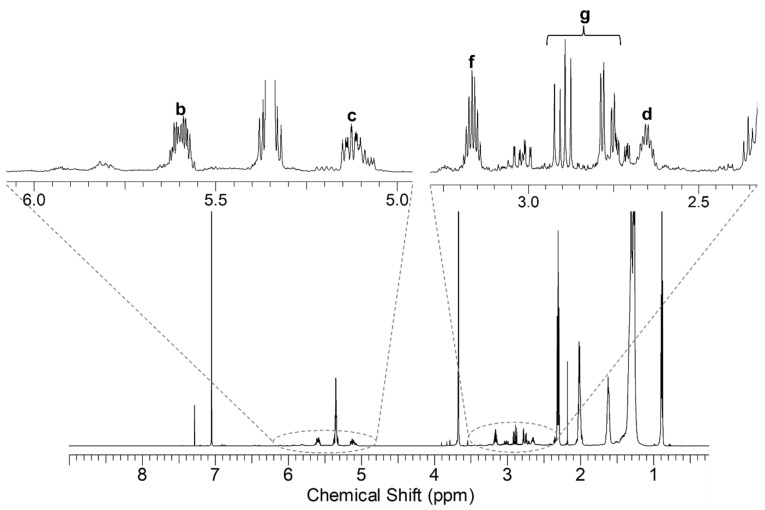
^1^H NMR spectrum of the reaction product of methyl oleate with MA (under MW activation). The protons of the alkenyl succinic anhydride group are labeled according to Figure 3.

The product of methyl oleate with MA contains the moiety shown in Figure 3, and the NMR spectrum (Figure 4) showed the following characteristic peaks (δ ^1^H ppm, δ ^13^C ppm): a (2.0, 32.9), b (5.61, 136.87), c (5.13, 126.66), d (2.65, 45.65), e (1.40, 33.03), f (3.16, 42.76), g (2.77–2.90, 30.34).

**Figure 5:**
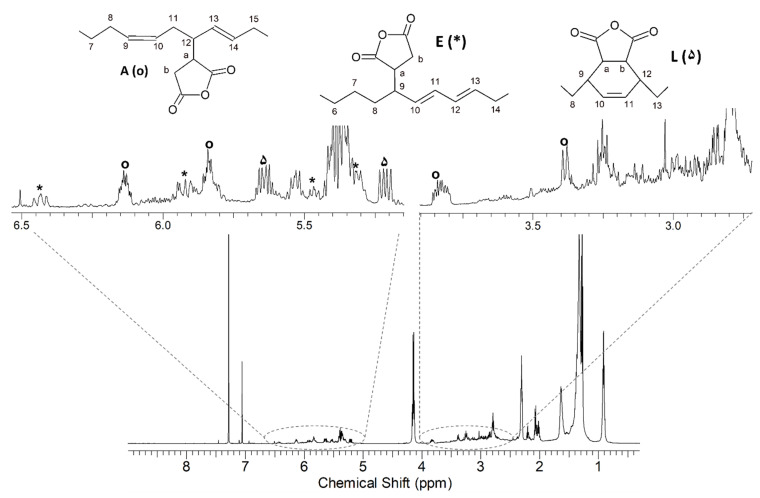
^1^H NMR spectrum of the reaction product of ethyl linoleate with MA; the signals are highlighted by different symbols: ۵ compound **A**; * compound **E** (or **F**); ο compound **L** (or **M**) of Scheme 3.

In the product of ethyl linoleate with MA, the three principal compounds found are shown with three different symbols in Figure 5.

The first is compound **A** of Scheme 3 (δ ^1^H ppm, δ ^13^C ppm): 8 (2.02, 32.63), 9 (5.53, 133.25), 10 (5.31, 125.68), 11 (2.20,), 12 (2.78, 42.7), a (3.25, 44.5), b (2.86–2.91, 33.12), 13 (5.21, 125.97), 14 (5.64, 136.78), 15 (2.02, 32.63), 16–17 (1.30, -), 18 (0.90, 14.1).

The compounds with conjugated double bonds (compounds **E** and **F** of Scheme 3) have the same pattern of chemical shifts in the ^1^H-NMR spectrum. The resonances of compound **E** (δ ^1^H ppm, δ ^13^C ppm) are reported here: 8 (2.51, -), 9 (2.78, 42.70), a (3.22, 43.70), b (2.76–2.92, 30.12), 10 (5.35, 128.20), 11 (6.43, 130.59), 12 (5.92, 127.65), 13 (5.46, 133.70), 14 (2.07, 27.30).

The compounds obtained by double bond isomerization and then by the Diels-Alder reaction are the compounds **L** and **M** of Scheme 3. These two isomers have the same values of chemical shift in the ^1^H-NMR spectrum. These are the resonances of compound **L** (δ ^1^H ppm, δ ^13^C ppm): 8 (2.34, 33.3), 9 (2.84, 37.3), 10 (6.14, 133.7), 11 (5.84, 129.8), 12 (2.99, 35.8), 13 (1.60, 35.9), a (3.38, 44.6), b (3.84, 43.4) [46].

If present, the double bond close to the ASA groups both in methyl oleate and in ethyl linoleate derivatives is in the trans configuration, in agreement with the data from the literature [33,47].

### 2.5. Reactions of Maleic Anhydride with Grapeseed, Hemp, and Linseed Oils

The reactions of MA with the vegetable oils produced complex mixtures of products, the appearance of which depended on the oil:MA ratio, the time of reaction, and the heating mode.

In the case of grapeseed oil, the reaction carried out by microwave activation for 30 min incorporated practically the same amount of MA as the reaction performed with conventional heating for 5 h, confirming also for this reaction a large reduction in time, even if it resulted in higher viscosity [33,48,49,50].

**Figure 6:**
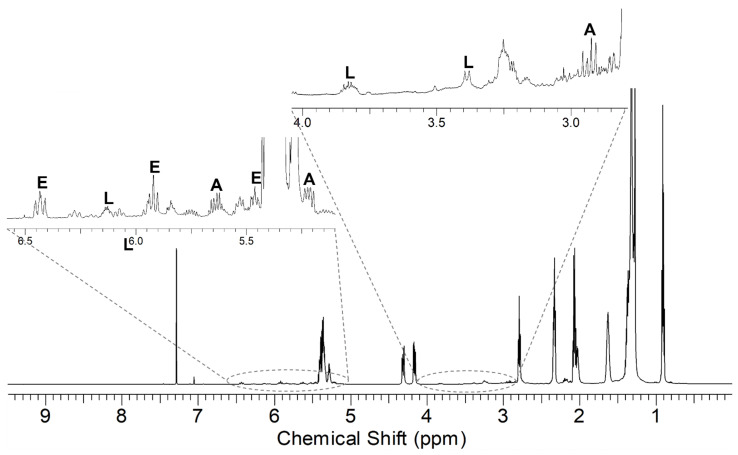
^1^H NMR of the sample GO2 MW; the signals of the main products refer to the compounds **A**, **E** (or **F**), and **L** (or **M**) of Scheme 3, bearing the same groups reported in Figure 5.

**Figure 7:**
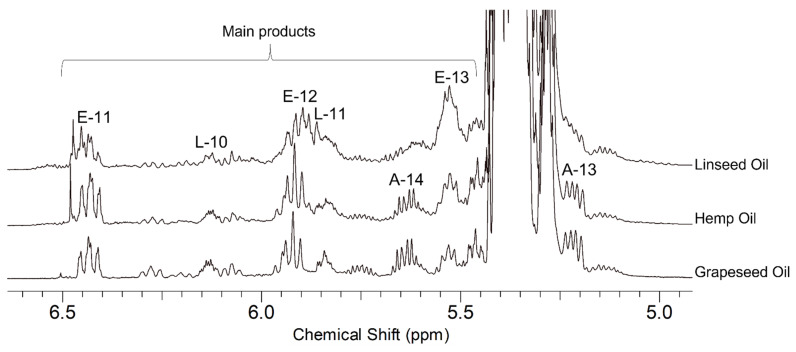
^1^H NMR spectral expansion of oil products with MA in the region of 6.6 -5.0 ppm. The signals have been attributed to the olefinic protons (numbers) of the adducts reported in Scheme 3 and Figure 5.

In Figure 6, the ^1^H NMR spectrum of the product from the reaction GO/MA, sample GO2 MW, is reported. The relevant region of the ^1^H NMR spectra of the three maleates GO, HO, and LO oils is shown in Figure 7. The signals of the ASA groups identified in the oleate and linoleate products were found in all cases. In hemp oil and linseed oil, the linolenic acid chain is also present; given the presence of three double bonds that could react with maleic anhydride, many more isomeric products are possible.

The mixtures obtained in the reactions of GO, HO, and LO with MA depended on the amount of MA, the reaction time, and the use of either thermal or microwave activation: specifically, a slightly higher degree of ASA substitution was achieved in all cases with microwave activation even though, in the latter case, the viscosity was higher. This result could be related to the occurrence of more efficient oligomerization processes in the sealed microwave reactor [40].

The viscosity of the samples measured through a CSR test was independent of the shear rate applied. All of the samples showed the typical trend of a Newtonian liquid in which the viscosity remains constant as the shear rate increases.

Compared to the viscosity of the starting oils measured at a shear rate of 0.1 s^−1^ (0.045 Pa·s for GO, 0.040 Pa·s for HO and LO), the functionalized oils showed an increase in viscosity depending on different factors: (i) *Oil/MA ratio*: comparing the GO2 MW and GO5 MW samples, the viscosity increased from 2.2713 Pa·s to values higher than 500 Pa·s.; (ii) *reaction time*: comparing GO2 MW and GO4 MW, the viscosity slightly increased from 2.2713 to 2.6555 Pa·s, and (iii) *the activation method*: for GO and HO, where small differences in the ASA mmol/g of oil were detected, the viscosity of the final mixtures obtained under conventional heating (for GO1 0.197 Pa·s and for HO1 1.448 Pa·s) was lower than that of GO 2MW (2.271 Pa·s) and HO1 MW (5.638 Pa·s). A similar trend was observed also for LO (0.233 Pa·s for LO1 and 23.427 Pa·s for LO MW) even though in LO1, the ASA content was significantly lower. These observations agree with the literature [41] where the increase in the viscosity is related to the increase in ASA content because of the disorder and the decreased alignment of chains and consequently a higher resistance of the fluids to flow.

**Figure 8:**
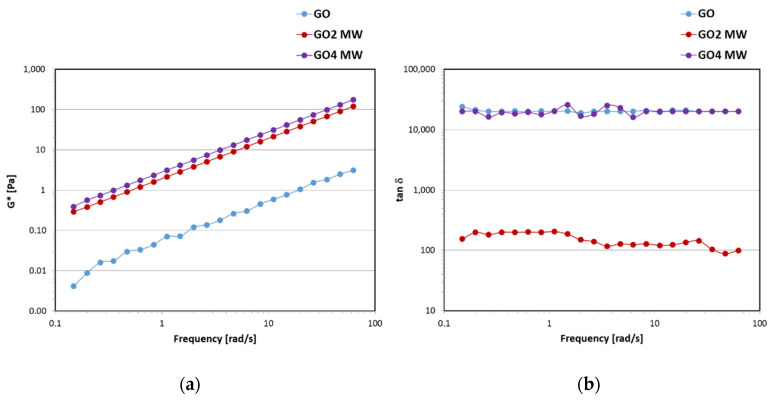
Trend of the complex G* modulus (**a**) and damping factor tanδ (**b**) in function of the oscillation frequency for GO, GO2 MW, and GO4 MW.

A preliminary investigation of the viscoelastic properties was carried out on the starting oil GO and on the functionalized mixtures GO2 MW and GO4 MW (Figure 8). The FS analyses, performed while maintaining the oscillation amplitude inside the LVER and decreasing the frequency, showed that the functionalized mixtures had higher values of G* modulus than the starting oil GO (Figure 8a), indicating an increase in the viscoelastic properties after functionalization. Between the samples GO2 MW and GO4 MW, no quantitative difference could be detected in the values of G*. For the samples under examination, the viscous modulus G’’ was always greater than the elastic modulus G’ in the whole range of deformation investigated, as shown by the tanδ values (Figure 8b). Tanδ is a rheological parameter calculated from the ratio between the G’’ and G’ modulus; values of tanδ > 1 correspond to liquid-like behavior in which the viscous component G’’ dominates over the elastic one G’ since the bonds between the individual molecules are weaker. GO2 MW showed the lowest values of tanδ, indicating a higher elastic G’ modulus than in GO4 MW and a greater degree of internal structuring.

The differences observed in the mixtures deriving from the reactions carried out on the oils between conventional heating and microwave activation could be ascribed to various effects specific to microwaves. (i) The first is the decreasing of the dielectric constant of the reaction mixture with time (a parameter that indicates the ability of a material to store electromagnetic energy by polarization) due to the consumption of MA, which has a high dielectric constant (52.57), in the oils with very low dielectric constants (≤ 3) [50]. (ii) The second is the higher or lower activation of reactions occurring through polar or nonpolar transition states. In the cases of isopolar transition state reactions such as Diels-Alder cycloaddition or ene reactions, no specific microwave effect would be expected even though it was observed in many cases [51], suggesting that polar transient species are involved in these processes. In the presence of a polar solvent or a polar reagent, such as MA, a strong coupling with the radiation can occur in the reaction mixture. This is particularly important in heterogeneous systems where microscopic hot spots or selective heating can be generated, thus explaining the higher yields in the somewhat lower times with respect to conventional heating. In any case, the activation energy reported for the reaction of fatty acid esters with an MA of about 77 kJ/mol [52] indicates that these processes (having activation energy higher than 20–30 kJ/mol, [50]) can be successfully performed under microwave irradiation. (iii) In the case of radical processes carried out by microwave activation, a “solvent cage-effect” (in our case an “oil cage-effect”) has also been proposed and studied, which occurs on the radical species formed during the reaction, thus limiting the collisions and their recombination [53].

All of these effects can act in a different manner on the different oils depending on the amount of linoleic and linolenic species, which can undergo Diels-Alder reactions, thus inducing a selectivity between the different mechanisms involved in the processes.

**Figure 9:**
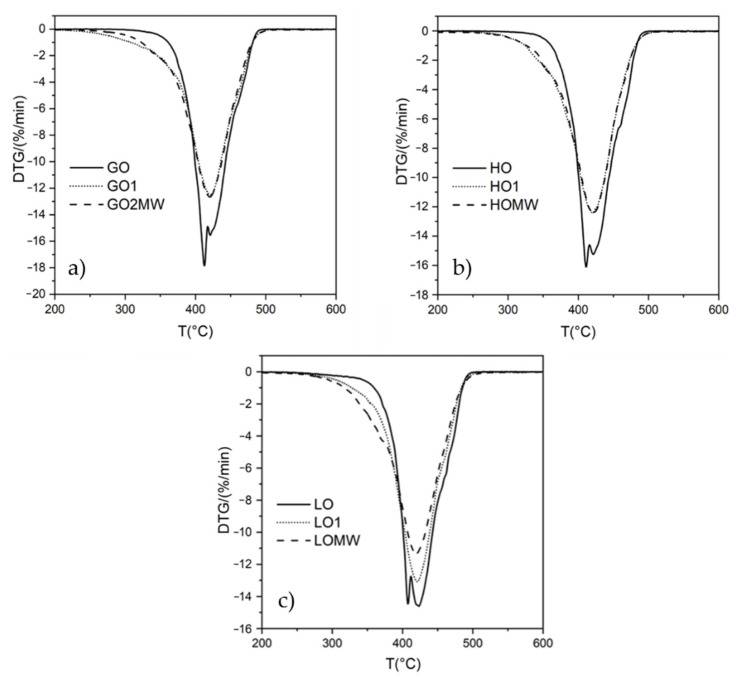
DTG curves for (**a**) GO, (**b**) HO, and (**c**) LO and the corresponding maleated derivatives.

To characterize the final mixtures, some thermogravimetric determinations were also carried out under nitrogen. The data are collected in Table 2. Figure 9 shows the TG and DTG curves of the oils and of the maleated derivatives. The degradation temperatures for pristine GO and HO are similar and higher compared to LO, considering both the onset temperature and the temperature at 5 wt% loss (Table 2). This is consistent with the highest content of linolenic acid in LO and with the general correlation between the number of double bonds and the reduced thermal stability.

All of the TGA profiles show similar trends (Figure S9), and both the onset temperature and the temperature at 5% weight loss of the maleated derivatives were lower with respect to the values of the starting oils, with negligible differences between the samples prepared by conventional heating or microwave activation. The DTG curves indicated two main overlapping steps for the oils because of the complex sequence of degradation processes involving the carbon–carbon cleavage with the formation of many species through radical mechanisms including olefins and polymers [54,55,56], probably occurring at the same time during thermal decomposition. For this reason, the identification of a single chemical species through the attribution of each decomposition step in the DTG profile is not trivial. However, for the maleate derivatives, an additional shoulder can be observed at lower temperatures as shown in Figure 9, which can be attributed to the degradation of the incorporated ASA moieties in the oil structure, while the main DTG peak could be due to the decomposition of the modified chains. On these bases, it is reasonable to deconvolute the DTG into three main components (Figure S10): two main steps at higher temperatures accounting for the thermal degradation of lipid and the derived polymers and one at lower temperatures for ASA moieties. The deconvolution of the DTG (Table S4) indicates the amounts of ASA moieties in the maleate derivatives which are in agreement with the titration data reported in Table 3.

The main decomposition rate of the modified oils is lower than their precursors, as reported in Table 2. This is consistent with the behavior reported by Gaglieri et al. [41]. The insertion of the anhydride moieties leads to the formation of complex reaction mixtures, as evidenced by the ^1^H and ^13^C NMR spectra where the substituted chains are more impeded and cannot properly align to a more ordered system, with a consequent increase in disorder leading to greater viscosity, as shown in this study. This is particularly true for LO and its derivatives where an MDR increase of 24% is observed for LOMW with respect to the pristine LO, which is to be expected considering the great increase in the measured viscosity and the ASA content reported here. 

## 3. Experimental Section

### 3.1. Materials and Methods

Maleic anhydride, methyl oleate, ethyl linoleate, and trans-3-octene were purchased from Merck and used as received. Grapeseed, hemp, and linseed oils were purchased in a local supermarket (all of the oils were of the Despar^®^ Vital brand, made in Italy).

#### 3.1.1. Reactions of Vegetable Oils and Maleic Anhydride Using Conventional Heating

The reactions carried out with conventional heating were performed under a nitrogen atmosphere in a three-neck round flask with a capacity of 250 mL, equipped with a heating mantle (LabHEAT KM-RX1001 with KM-G2-250 mL, SAF Wärmetechnik GmbH, Mörlenbach, Germany) and a magnetic stirrer. A condenser heated at 60 °C was connected to the central neck to control the MA sublimation; the nitrogen inlet was connected in the second neck, and the third neck was used to add MA and for sample collection during the reactions. The reaction mixtures were heated to 200 °C (heating rate 5 °C/min). Over time, the reaction mixtures became progressively brown, and viscosity increased. The reactions were followed by FTIR collecting samples every hour and stopped after 5 h. The reaction mixtures were cooled and subjected to centrifugation at 10,000 rpm for 10 min to separate the unreacted MA, if present. The experimental details are summarized in Table 3. Experiments were performed in triplicate and the data were averaged.

#### 3.1.2. Reaction of Methyl Oleate with Maleic Anhydride Using Conventional Heating

A total of 50.01 g (0.17 mol) of Methyl oleate (MO) was allowed to react with an equimolar amount of MA (16.50 g) in a three-necked round flask with a capacity of 250 mL and heated at 200 °C (heating rate 5 °C/min) as described above. The reaction was stopped after 5 h, collecting samples every half hour to record FTIR spectra. Only after 3 h at 200 °C did the FTIR spectrum clearly indicate ASA formation (absorptions at 918 and 1861 cm^−1^, with a significant decrease in the absorption at 696 cm^−1^ of free MA). The reaction mixtures were cooled and subjected to centrifugation at 10,000 rpm for 10 min to separate the unreacted MA. The yield was 28% as determined by ^1^H NMR integration and 26% by titration. A brown viscous liquid had formed. ESI (MO = C_19_H_36_O_2_, MW 296.49; MA = C_4_H_2_O_3_, MW 98.06): *m*/*z* 417 [MO + MA + Na]^+^ (100%).

#### 3.1.3. Reactions of Vegetable Oils and Maleic Anhydride by Microwave Irradiation

The reactions carried out by microwave irradiation were performed in an Anton Paar Monowave 200 Microwave reactor (with a nominal power of 850 W, Anton Paar GmbH, Graz, Germany) using a glass reactor (10 mL), which was connected to temperature and pressure controllers. The system was heated at a rate of 10 °C/min until the final temperature of 230 °C; then it was held for steps of 15 min to collect samples and record the FTIR spectra. The experimental details are summarized in Table 3. Experiments were performed in triplicate and the data were averaged.

#### 3.1.4. Reaction of Trans-3-Octene with Maleic Anhydride by Microwave Irradiation

A volume of 3 mL of trans-3-octene (2.15 g, 19.14 mmol) was allowed to react with an equimolar amount of MA (1.88 g) in the Anton Paar Monowave 200 at 230 °C for steps of 15 min. After 15 min, the FTIR spectra showed the absorption at 1861 cm^−1^ of the ASA moiety, which had become more intense after 1 h when the reaction was stopped. A viscous yellow liquid had formed. The yield was 82% by ^1^H NMR integration and 80% by titration. ESI (T3O = C_8_H_16_, MW 112.21; MA = C_4_H_2_O_3_, MW 98.06): *m*/*z* 477 [2T3O + 3MA-CO_2_ + 3H]^+^ (40%); *m*/*z* 605 [3T3O + 3MA-CO_2_ + 3H + CH_3_]^+^ (100%).

#### 3.1.5. Reaction of Methyl Oleate with Maleic Anhydride by Microwave Irradiation

A volume of 3 mL of methyl oleate (2.62 g, 8.84 mmol) was allowed to react with an equimolar amount of MA (0.87 g) in the Anton Paar Monowave 200 at 230 °C for steps of 15 min and stopped after 1 h. The reaction mixture was cooled and subjected to centrifugation at 10,000 rpm for 10 min to separate the unreacted MA. After 30 min at 230 °C, the FTIR spectrum clearly indicated ASA formation. The yield was 60 % by ^1^H NMR integration and 57% by titration. A light brown viscous liquid formed. ESI (MO = C_19_H_36_O_2_, MW 296.49; MA = C_4_H_2_O_3_, MW 98.06): *m*/*z* 417 [MO + MA + Na]^+^ (100%).

#### 3.1.6. Reaction of Ethyl Linoleate with Maleic Anhydride by Microwave Irradiation

A volume of 3 mL of ethyl linoleate (2.63 g, 8.52 mmol) was allowed to react with an equimolar amount of MA (0.83 g) in the Anton Paar Monowave 200 at 230 °C for steps of 15 min. The reaction was stopped after 1 h. The reaction mixture was cooled and subjected to centrifugation at 10,000 rpm for 10 min to separate the unreacted MA. After 30 min at 230 °C, the FTIR spectrum clearly indicated ASA formation. An orange viscous liquid formed. Yield 33% (by ^1^H NMR integration; 32% by titration). ESI (EL = C_20_H_36_O_2_, MW 308.50; MA = C_4_H_2_O_3_, MW 98.06): *m*/*z* 429 [EL + MA + Na]^+^ (58%); *m*/*z* 445 [EL + MA + K]^+^ (100%).

### 3.2. Characterization Techniques

The starting materials (trans-3-octene, ethyl linoleate, methyl oleate, grapeseed oil, hemp oil, and linseed oil), used as received, were characterized by multinuclear NMR experiments using a 600 MHz Bruker Avance spectrometer equipped with a Prodigy cryoprobe. Furthermore, ^1^H and ^13^C assignments of each product were determined by 1D conventional and CSSF-TOCSY [48] experiments with the support of 2D COSY, HSQC, and HMBC experiments.

Fourier Infrared Spectroscopy (FTIR) analyses were recorded with a Perkin-Elmer Spectrum 100 using NaCl plates as support for all of the samples in the range of 4000–400 cm^−1^.

The amount of succinic anhydride formed during the reactions was determined by titration with NaOH and phenolphthalein: about 1 g of adduct was put into a flask, 20 mL of acetone was added, the solution was stirred until the solubilization of the adduct; then 0.4 mL of distilled water was added, and the mixture was kept for 15 min at 60 °C to hydrolyze the succinic anhydride. The acid formed was titrated with a NaOH solution (0.2 mol/L) in water. To determine the average number of ASA units incorporated per triglyceride, we considered that each anhydride unit consumes 2 equivalents of base, and the base amounts due to the determination of the soap numbers of the starting oils were subtracted [33,40].

The ESI MS analyses were performed using a Finningan LCQ-Duo ion-trap instrument (Thermo Fischer Scientific, Milan, Italy), operating in positive ion mode (sheath gas flow N_2_ 30 a.u., source voltage 4.0 kV, capillary voltage 21 V, capillary temperature 200 °C). The He pressure inside the trap was kept constant. The pressure, directly read by an ion gauge (in the absence of the N_2_ stream), was 1.33 × 10^−5^ Torr. Sample solutions were prepared by dissolving the compounds (3 mg) in CH_3_CN (5 mL) and diluting 1 mL of the solution in CH_3_CN (5 mL) immediately before analysis.

Thermogravimetric analysis of the final products was carried out on 19–20 mg of samples placed on Al_2_O_3_ crucibles under a N_2_ atmosphere (60 mL/min), heating from RT to 650 °C at 10 °C/min using a Netzsch STA 449 C analyzer. Data were processed and analyzed by Netzsch Proteus software, and the deconvolution of the DTG curves was calculated by the Microcal Origin Pro fitting routine.

Rheological determinations were performed with an Anton-Paar rheometer Physica MCR e302 equipped with a con-plate CP50-P1 geometry with a fixed gap of 0.098 mm. The analyses were conducted both in continuous and oscillatory flow conditions, setting the temperature at 23 °C ± 0.05 °C. A Controlled Shear Rate (CSR) test was conducted to determine the viscosity values (η) and the flow behavior of the samples at an increasing shear rate, ranging from 0.001 to 1000 s^−1^. The samples’ viscoelastic properties, i.e., the trend of storage elastic (G’) and loss viscous (G’’) moduli, were evaluated through tests in an oscillatory regime conducted at small deformations where the upper plate oscillated, and the lower plate remained stationary. Amplitude sweep (AS) tests were performed fixing the frequency at 1 Hz while increasing the strain values (γ) from 0.01% to 1000% to identify the samples’ linear viscoelastic region (LVER). In this region, both the elastic and the viscous moduli remained constant until critical strain (γ G’ = G’’), indicating the strain range in which the measurements can be carried out without irreversibly altering the structure of the product. Selecting a percentage γ value inside the LVER and keeping it fixed, frequency sweep (FS) tests were performed varying the oscillation frequency from 10 Hz to 0.01 Hz to analyze the samples’ inner structure and the type of intermolecular interactions that characterize the materials expressed by the complex modulus (G*) and the damping factor (tanδ) [57,58]. G* is defined as the ratio between the shear stress and the shear strain under oscillatory conditions. Tanδ is calculated from the ratio between the G’’ and G’ moduli.

### 3.3. DFT-NMR Calculations

A conformational search for each compound investigated by Density Functional Theory (see Section 2) was run with SPARTAN02 using the MMFF94 Force Field [59]. The conformers were then submitted for energy minimization at the B3LYP/6-31 + G(d) level. The lowest energy conformers with energy within 2 kcal/mol (accounting for more than 96% of the population at 298 K) from the most stable one, were selected for the GIAO-B3LYP/pcS-2 [60] calculation of the isotropic shielding constant, σ, following established protocols [61]. The shielding constant of TMS at the same level of theory was used to calculate the chemical shift as δ = σ_ref_ − σ. Chemical shifts were then averaged based on the Boltzmann population of each conformer. DFT calculations were run using the software package Gaussian 16 [62].

## 4. Conclusions

The reactions of grapeseed, hempseed, and linseed oils with maleic anhydride were studied with the objective of attaining the functionalization of the hydrophobic oils with a polar reactive alkenyl succinic anhydride group to promote further reactions for the achievement of water dispersions for industrial applications. The reactions occur through different mechanisms yielding complex mixtures of unsaturated and saturated products depending on the presence of one or more double bonds in the starting materials. To clearly identify the most relevant products formed, trans-3-octene, methyl oleate, and ethyl linoleate were also reacted. The reactions were carried out either by conventional heating or by microwave activation. Through a detailed NMR investigation, the products formed in the reaction of trans-3-octene were identified, and the results were supported by a computational study. The signals of the alkenyl succinic anhydride (ASA) moiety bound to the methyl oleate and ethyl linoleate chains were identified, thus allowing us to recognize them in the quite complex spectra of the oil derivatives. The reactions carried out under conventional heating yielded a similar or lower amount of ASA groups bound to the organic chains with respect to the reactions carried out under microwave activation, which required very short times. However, in the latter case, the viscosity of the reaction mixtures was higher. In all cases, an increase in viscosity with time after the reactions was observed; this effect appeared particularly evident in the case of microwave reactions. This aspect deserves further investigation to better understand the specific effect of microwave activation, at least in this type of reaction.

## Figures and Tables

**Table 1:** Chemical shifts (multiplicity) of trans-3-octene’s products according to the labeling of Scheme 4. d = doublet, t = triplet, m = multiplet, s = septet, dd = doublet of doublets, qd = quartet of doublets, dt = doublet of triplets, qt = quintet.

Species	A		A’		B		C	
δ [ppm]	δ ^1^H	δ ^13^C	δ ^1^H	δ ^13^C	δ ^1^H	δ ^13^C	δ ^1^H	δ ^13^C
1	1.67 (dd)	18.1	1.66 (dd)	13.4	0.90 (t)	11.9	0.90 (t)	11.9
2	5.60 (dd)	130.9	5.61 (dd)	130.9	1.61 1.56 (m)	25.2	1.97 (qd)	34.7
3	5.10 (dd)	127.1	5.15 (dd)	128.4	2.22 (s)	47.1	5.59 (dt)	136.6
4	2.32 (s)	45.0	2.62 (s)	42.7	5.07 (dd)	127.3	5.12 (dd)	126.6
5	1.58 1.48 (m)	25.1	1.38 (m)	25.40	5.59 (dd)	136.7	2.54 (s)	44.6
6	1.29 (m)	22.4	1.29 (m)	22.4	1.99 (m)	34.7	1.42 1.50 (m)	25.4
7	1.20 (m)	22.4	1.21 (m)	22.4	1.36 (m)	22.5	1.35 (m)	32.1
8	0.88 (t)	13.9	0.88 (t)	13.9	0.87 (t)	13.9	0.85 (t)	13.8
a	3.17 (m)	45.2	3.16 (m)	45.1	3.19 (m)	45.1	3.18 (qt)	45.11
b, b’	2.71 3.03 (dd)	32.7	2.74 2.90 (dd)	30.2	2.71 3.03 (dd)	32.7	2.75 2.90 (dd)	30.3

**Table 2:** TGA under N_2_ and DTG data for GO, HO, LO, and related maleinization products.

Sample	TGA	TGA	Residual Mass @650 °C	DTG (1)	DTG (2)	DTG (1)	DTG (2)
	T °C Onset	T °C @5%wt Loss	%	T °C	T °C	MDR (mg/min^−1^)	MDR (mg/min^−1^)
**GO**	390.4	378.3	0.4	412.7	420.9	−4.4	−3.8
GO 1	373.7	316.4	1.8	-	421.5	−	−3.6
GO 2 MW	375.2	336.2	2.93	-	420.7	−	−3.3
**HO**	389.9	378.4	0.55	411.2	421.4	−4.1	−3.9
HO 1	375.7	339.5	3.18	-	420.4	−	−2.9
HO MW	375.4	334	3.09	-	421.6	−	−3.2
**LO**	386.7	370.9	0.93	407.7	423.3	−4.1	−4.2
LO 1	379.9	341	2.18	-	420.9	−	−3.8
LO MW	369.7	328.1	4.28	-	419.13	−	−3.2

**Table 3:** Experimental details and some properties of the reaction products between the vegetable oils and maleic anhydride.

Vegetable Oil	Sample Labeling	Experimental Details	Aspect	ASA mmol/g of Oil	Viscosity, η (Pa•s)
Grapeseed oil, GO	GO	-	uncolored	-	0.045
	GO 1	Oil/MA = 50 g/9.3 g; 200 °C; 5 h	pale yellow	0.85 ± 0.03	0.197 ^a^ 7.574 ^b^
	GO 2	Oil/MA = 50 g/20 g; 200 °C; 5 h	brown	-	>500 ^a^
	GO 3	Oil/MA = 50 g/30 g; 200 °C; 12 h	brown	1.99 ± 0.06	167.79 ^b^
	GO 4	Oil/MA = 50 g/0 g; 200 °C; 5 h	dark brown	-	30.802 ^b^
	GO 1 MW	Oil/MA = 50 g/9.3 g; 1 × 15 min	pale yellow	-	-
	GO 2 MW	Oil/MA = 50 g/9.3 g; 2 × 15 min	yellow	0.92 ± 0.03	2.271 ^a^ 15.403 ^b^
	GO 3 MW	Oil/MA = 50 g/9.3 g; 3 × 15 min	yellow	-	-
	GO 4 MW	Oil/MA = 50 g/9.3 g; 4 × 15 min	yellow	0.91 ± 0.03	2.656 ^a^
	GO 5 MW	Oil/MA = 50 g/30 g; 2 × 15 min	brown	-	>500 ^a^
	GO 6 MW	Oil/MA = 50 g/20 g; 2 × 15 min	light brown	1.88 ± 0.05	>500 ^a^
Hemp oil, HO	HO	-	pale yellow	-	0.04
	HO 1	Oil/MA = 50 g/9.3 g; 200 °C; 5 h	yellow	0.79 ± 0.03	1.448 ^a^
	HO MW	Oil/MA = 50 g/9.3 g; 2 × 15 min	yellow	1.01 ± 003	5.638 ^a^
Linseed oil, LO	LO	-	yellow	-	0.040
	LO 1	Oil/MA = 50 g/9.3 g; 200 °C; 5 h	yellow	0.43 ± 0.02	0.233 ^a^
	LO MW	Oil/MA = 50 g/9.3 g; 2 × 15 min	orange	1.23 ± 0.04	23.427 ^a^

^a^ The viscosity measurements were carried out within a week after preparation. ^b^ The viscosity measurements were carried out after 6 months after preparation.

## Data Availability

Not applicable.

## Supplementary Materials

The following supporting information can be downloaded at: https://www.mdpi.com/article/10.3390/molecules27238142/s1, Table S1: ^1^H and ^13^C assignments of trans-3-octene (98% purity); Figure S1: Structure and ^1^H NMR spectrum of trans-3-octene; Figure S2: ^1^H NMR spectrum of methyl-oleate; Figure S3: ^1^H NMR spectrum of ethyl-linoleate; Table S2: NMR data and assignments for oleic acid, linoleic acid, and linolenic acid; Table S3: Composition of grapeseed oil, hemp oil, and linseed oil; Figure S4: HMBC spectrum of the final reaction mixture of trans-3-octene and maleic anhydride; Figure S5: HSQC spectrum of the final reaction mixture of trans-3-octene and maleic anhydride; Figure S6: HSQC of the final reaction mixture of grapeseed oil and maleic anhydride; Figure S7: HMBC of the final reaction mixture of grapeseed oil and maleic anhydride; Figure S8: Correlation coefficients, R^2^, obtained from the linear fitting of calculated vs. experimental ^13^C chemical shifts, and Corrected Mean Absolute Errors (CMAE) for the same correlations; Figure S9: TGA profiles for the GO, HO, and LO and the corresponding maleate derivatives; Figure S10: Deconvolution of the derivatives of mass loss with respect to temperature for maleinization derivatives; Table S4: Area % calculation for GO1, GO2MW, HO1, HOMW, LO1, and LOMW from the deconvolution of the DTG curves into three components.
