# Supplementary material for: Activation of Vegetable Oils by Reaction with Maleic Anhydride as a Renewable Source in Chemical Processes: New Experimental and Computational NMR Evidence
Source: Molecules. 2022 Nov 23;27(23):8142. doi: 10.3390/molecules27238142 (PMC9741123; doi:10.3390/molecules27238142)
Supplement: Supplementary file 1 [file molecules-27-08142-s001.zip › molecules-2042586-supplementary revised.pdf]

SUPPLEMENTARY INFORMATION

The Activation of Vegetable Oils by Reaction with Maleic Anhydride as a Renewable Source in Chemical Processes: new experimental and computational NMR evidences

Francesco Lanero, Bianca Maria Bresolin, Anna Scettri, Marco Nogarole, Elisabetta Schievano, Stefano Mammi, Giacomo Saielli, Alessia Famengo, Alessandra Semenzato, Giovanni Tafuro, Paolo Sgarbossa, Roberta Bertani

TABLE S1. ^{1}H and ^{13}C assignments of trans-3-octene (98% purity).

Nucleus	Functional Group	Trans-3-Octene	
		δ ^{1}H (ppm)	δ ^{13}C (ppm)
1	$-CH_2$-**CH_3**	1.00	14.1
2	=CH-**CH_2**-CH_3	2.03	25.7
3	=**CH**-CH_2-	5.47	131.9
4	=**CH**-CH_2-	5.42	129.5
5	=CH-**CH_2-**	2.01	32.4
6	**-CH_2-**	1.36	31.9
7	**-CH_2-**	1.35	22.4
8	$-CH_2$-**CH_3**	0.93	14.1

FIGURE S1. Structure and ^{1}H NMR spectrum of trans-3-octene.

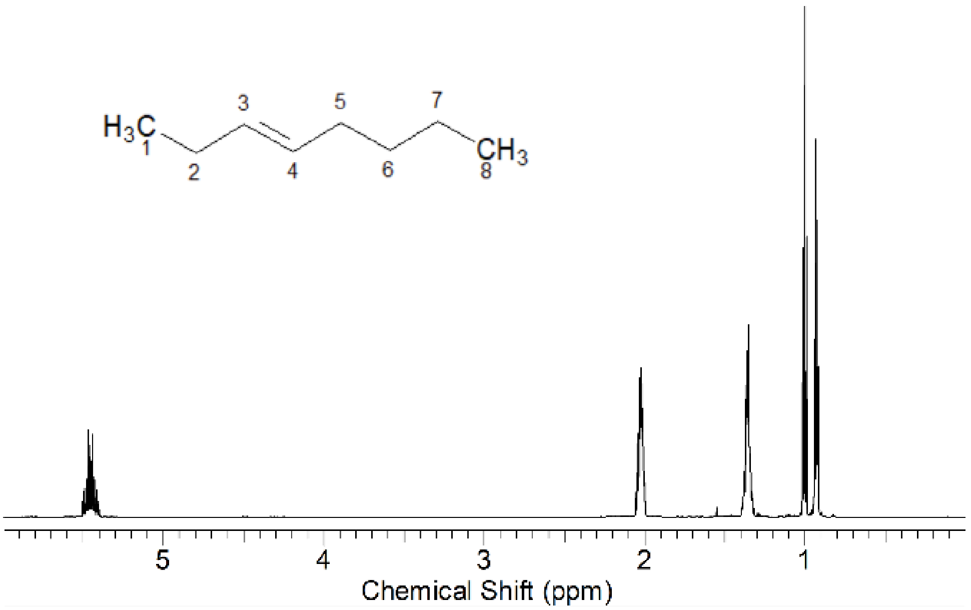

FIGURE S2. ^{1}H NMR spectrum of methyl-oleate. The purity was 85% due to the presence of linoleate as demonstrated by the specific signal of bis-allylic protons.

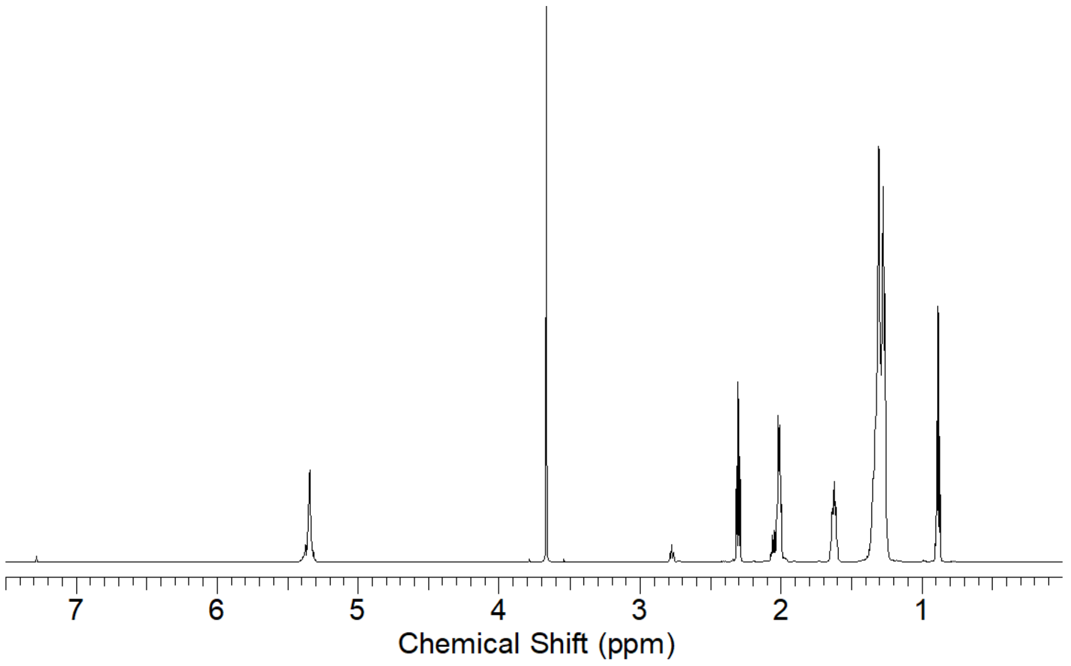

FIGURE S3. ^{1}H NMR spectrum of ethyl-linoleate (93% purity).

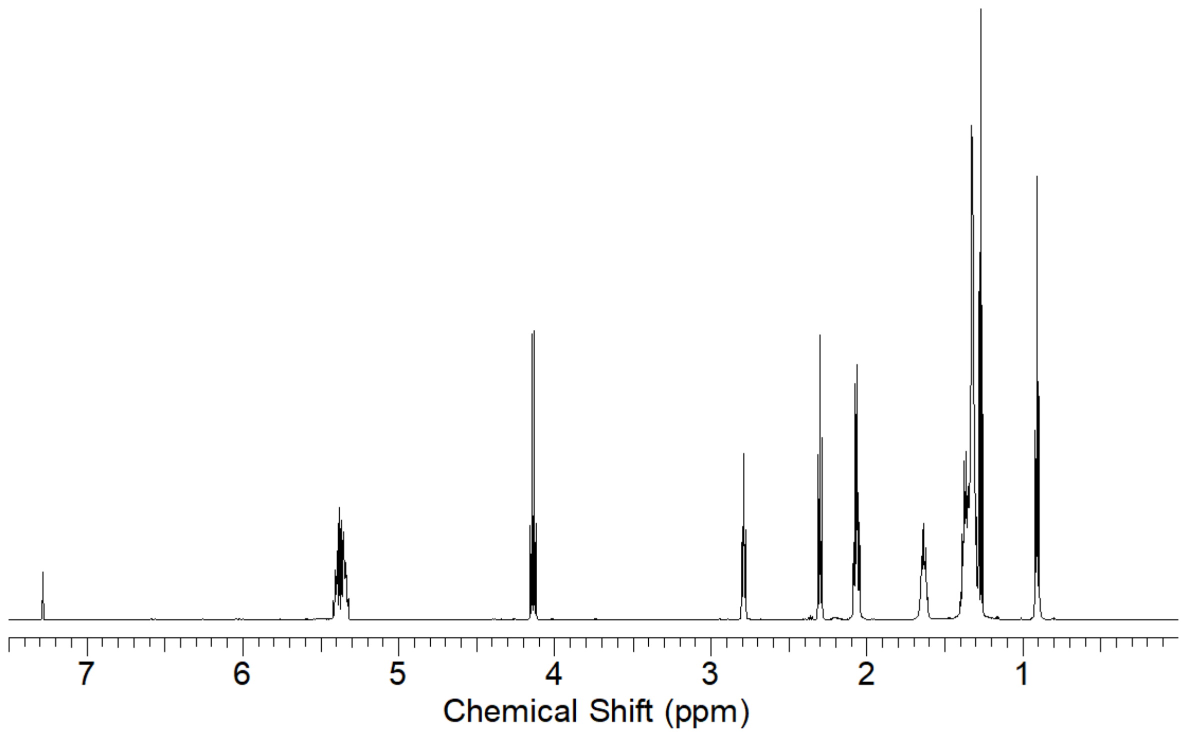

TABLE S2. NMR data and assignments for oleic acid, linoleic acid, and linolenic acid.

Nucleus Name	Functional Group	Oleic Acid		Linoleic Acid		Linolenic Acid	
		δ ^{1}H (ppm)	δ ^{13}C (ppm)	δ ^{1}H (ppm)	δ ^{13}C (ppm)	δ ^{1}H (ppm)	δ ^{13}C (ppm)
A	=CH-**CH₂**-CH=	-	-	2.78	25.84	2.80	25.70
B	-COOH-**CH₂**-CH₂	2.30	34.07	2.32	34.56	2.35	34.18
C	=CH-**CH₂**-	2.00	27.15	2.06	27.36	2.04	27.30
D	-COOH-CH₂-**CH₂**-	1.62	24.92	1.63	25.16	1.61	24.97
E	-CH₂-**CH₃**	0.88	14.06	0.90	14.24	0.96	14.41
F	**-CH₂-**	1.30	22.6-31.8	1.30	22.7, 29.34, 29.73	1.31	22-29
G	=**CH**-CH₂-	5.34	129.70, 129.96	5.37	128.20, 130.31	5.36	128.14, 130.02

The composition as percentage values of the three oils are shown in the following Table SI-3 as determined through ^{1}H-NMR spectra by the integration of proton's specific signal and by using simple equations described by Y. Zhang et al. J.Food Compos Anal, 2018, 69, 140-148. doi.org/10.1016/j.jfca.2018.03.006

$$[Linolenic\ Acid] = \frac{I_{0.96}}{I_{0.96} + I_{0.88}} \quad (1)$$

$$[Linoleic\ Acid] = \frac{3 \cdot I_{2.78} - 4 \cdot I_{0.96}}{2 \cdot (I_{0.96} + I_{0.88})} \quad (2)$$

$$[Oleic\ Acid] = \frac{3 \cdot I_{2.03}}{4 \cdot (I_{0.96} + I_{0.88})} - [Linolenic\ a.] - [Linoleic\ a.] \quad (3)$$

$$[Saturated\ Fatty\ Acids] = \frac{I_{0.88}}{I_{0.96} + I_{0.88}} - [Linoleic\ a.] - [Oleic\ a.] \quad (4)$$

TABLE S3. Composition of grapeseed oil, hemp oil, and linseed oil.

	Linolenic Acid	Linoleic Acid	Oleic Acid	Saturated Fatty Acids
Grapeseed Oil	1	70	19	10
Hemp Oil	18	57	13	12
Linseed Oil	51	18	21	10

FIGURE S4: HMBC spectrum of the final reaction mixture of trans-3-octene and maleic anhydride.

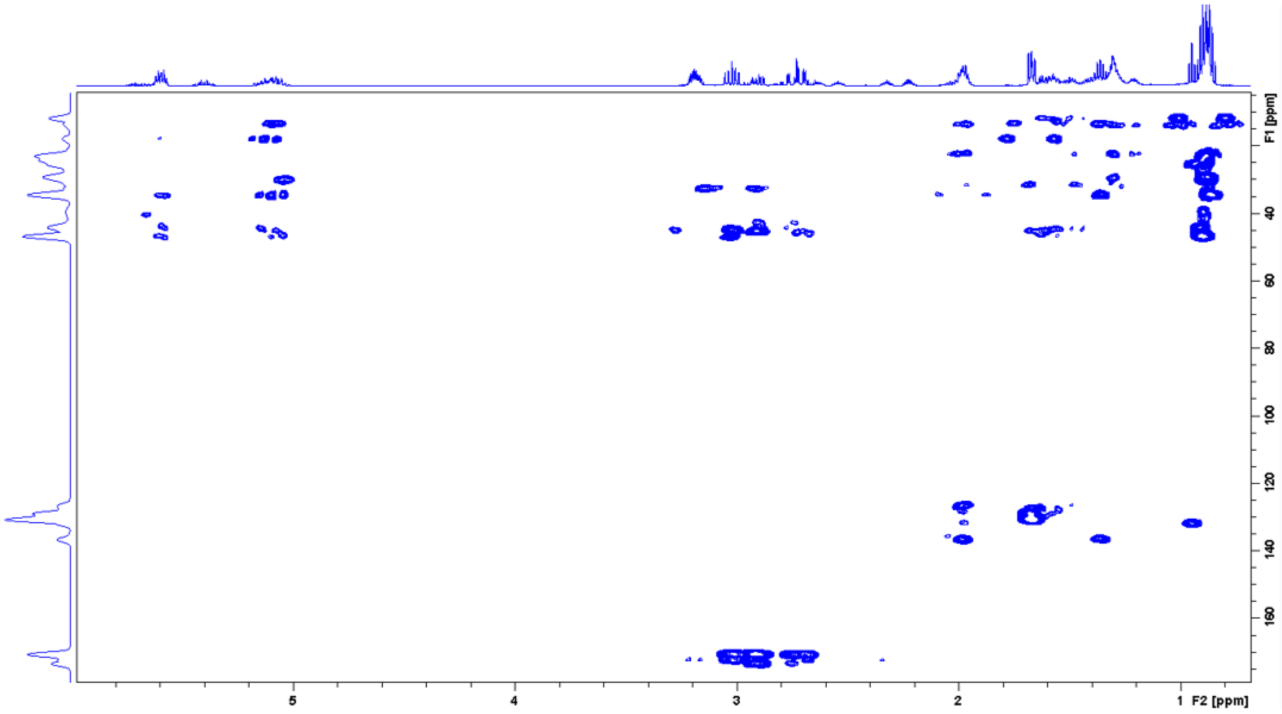

FIGURE S5: HSQC spectrum of the final reaction mixture of trans-3-octene and maleic anhydride.

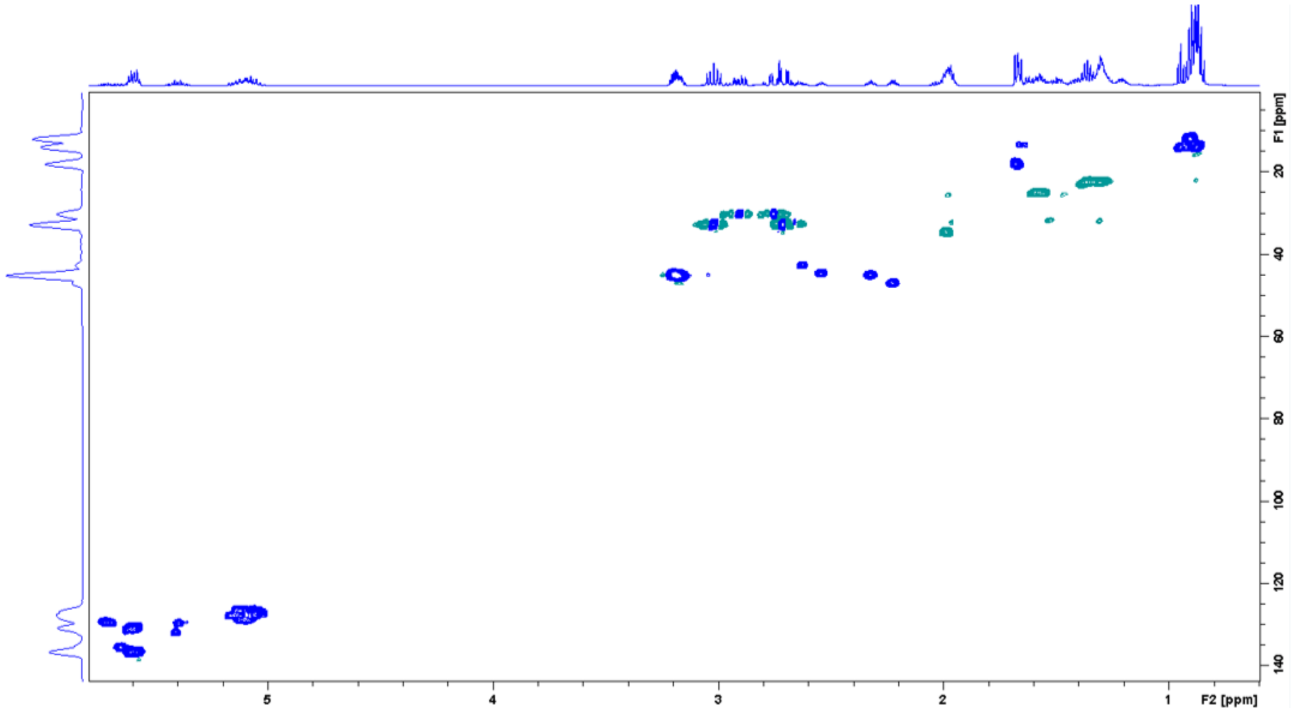

FIGURE S6: HSQC of the final reaction mixture of grapeseed oil and maleic anhydride.

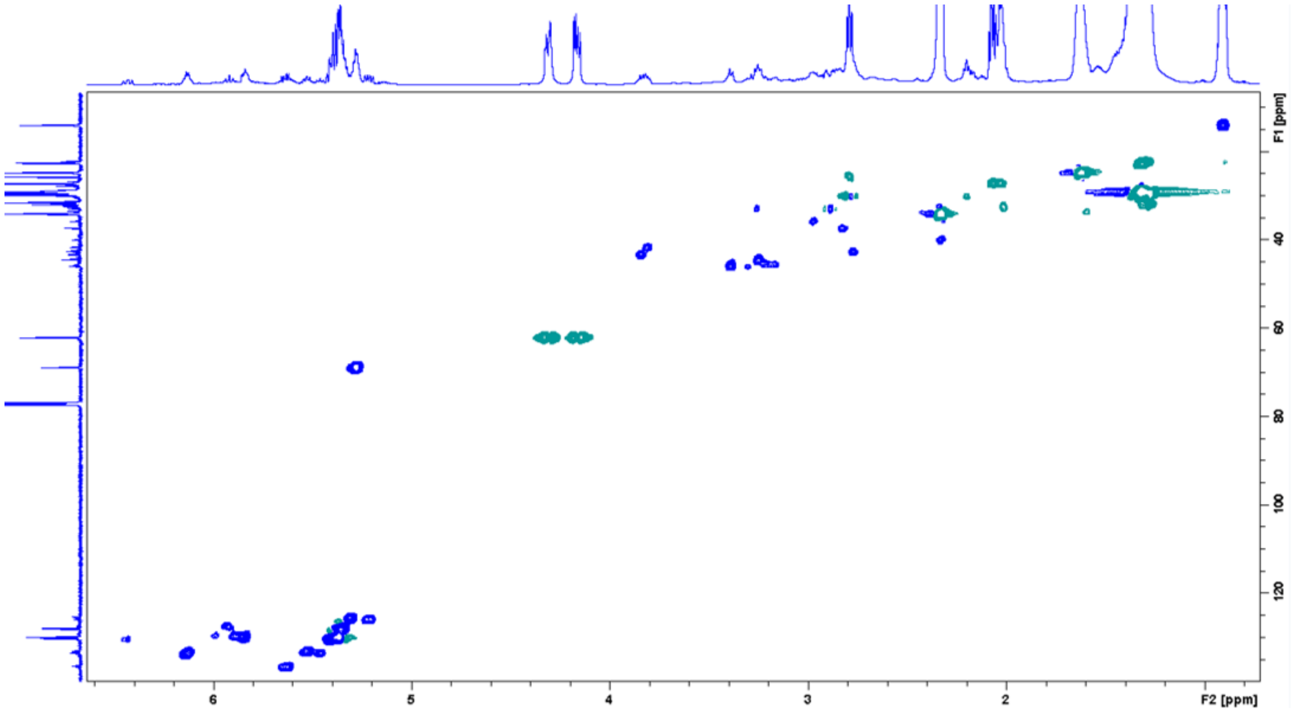

FIGURE S7: HMBC of the final reaction mixture of grapeseed oil and maleic anhydride.

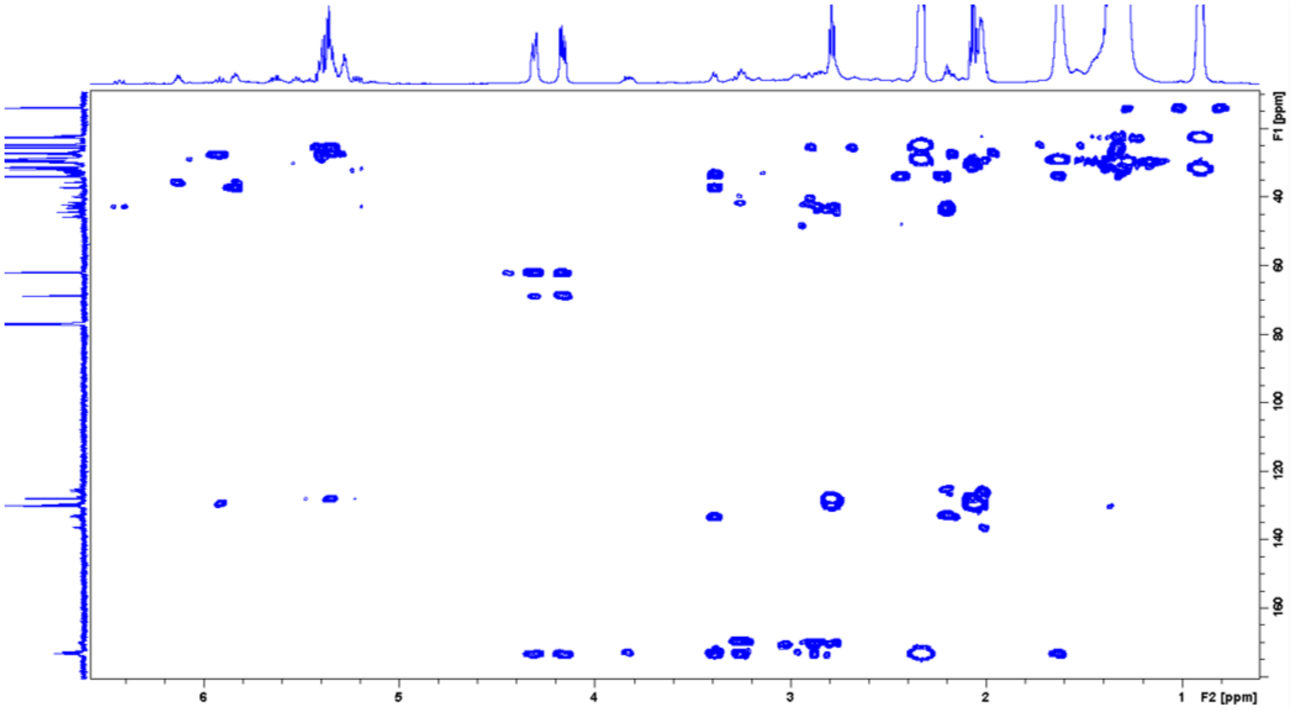

FIGURE S8. (Top) Correlation coefficients, R^2, obtained from the linear fitting of calculated vs experimental ^{13}C chemical shifts. For each molecule tested, **a**, **b**, **c** and **d**, we have two diastereoisomers, (S,S) and (S,R), where the first label refers to the chain carbon while the second one to the succinic anhydride carbon. Each calculated set is then correlated with the four experimental sets of ^{13}C resonances, labelled **a**, **a'**, **b**, **c** in Table 2. (Bottom): Corrected Mean Absolute Errors (CMAE) for the same correlations.

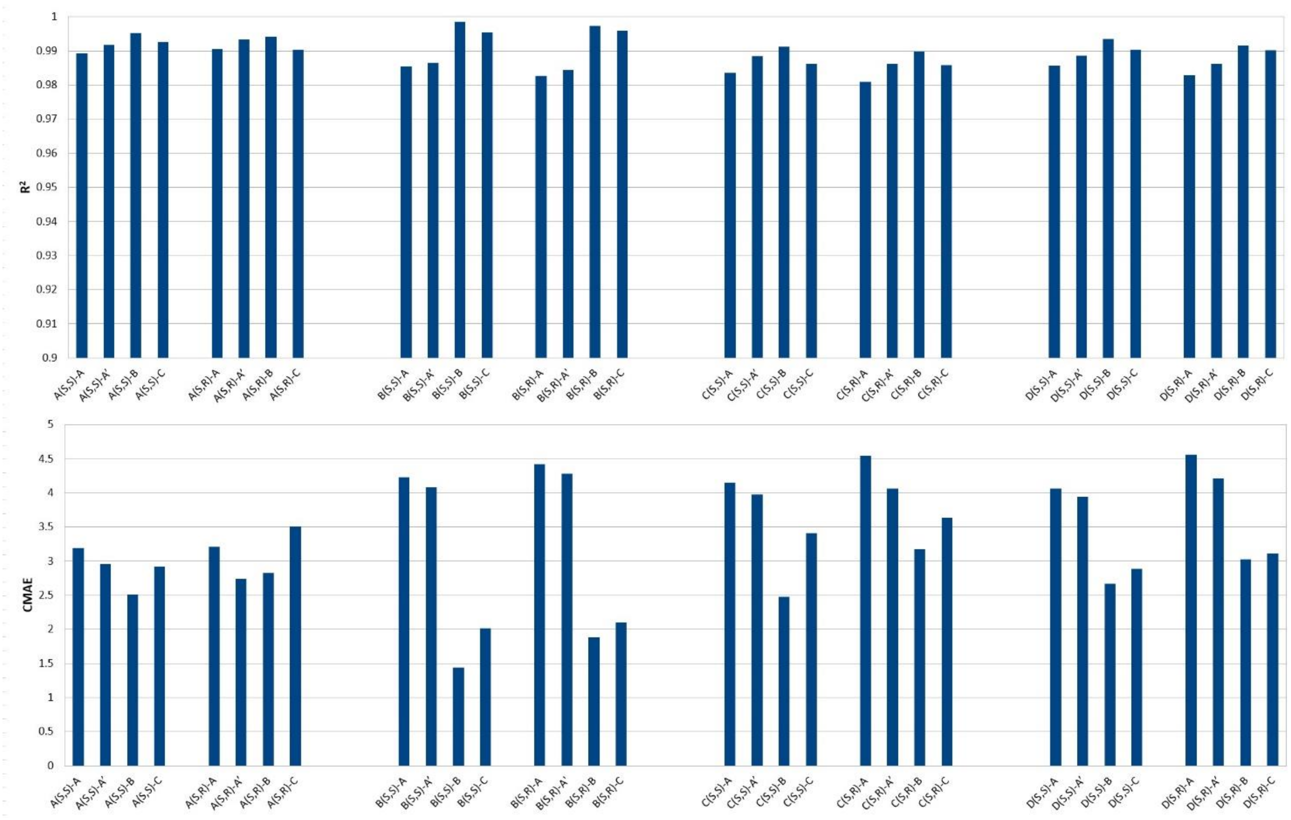

FIGURE S9: TGA profiles for the GO, HO, and LO and the corresponding maleate derivatives.

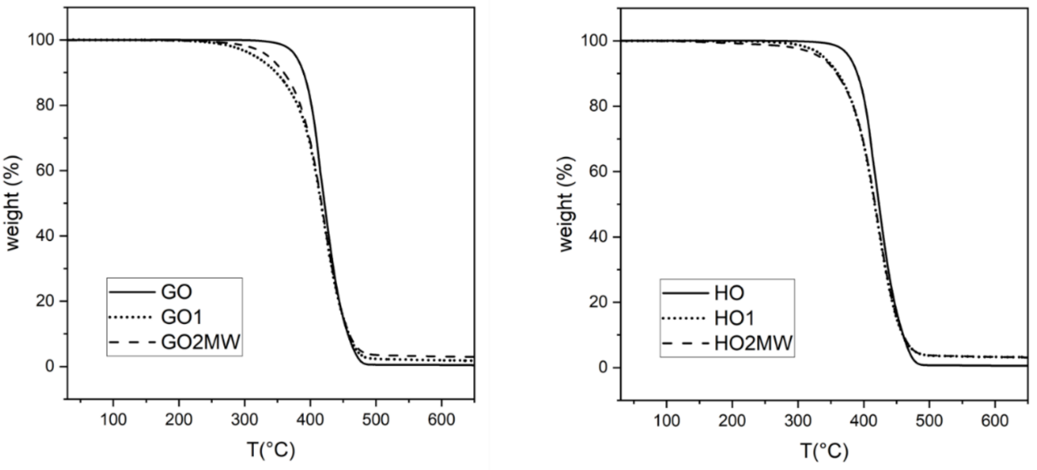

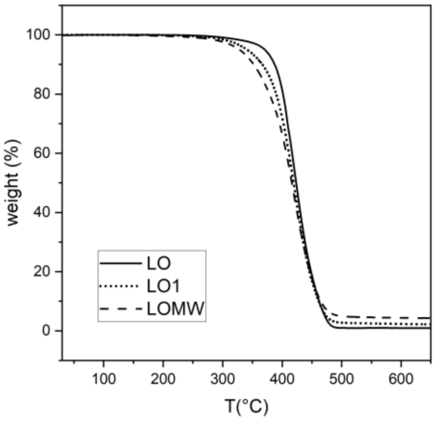

FIGURE S10 Deconvolution of the derivatives of mass loss with respect to temperature for maleinization derivatives. Red: experimental DTG. See TABLE SI-4 for fitting models

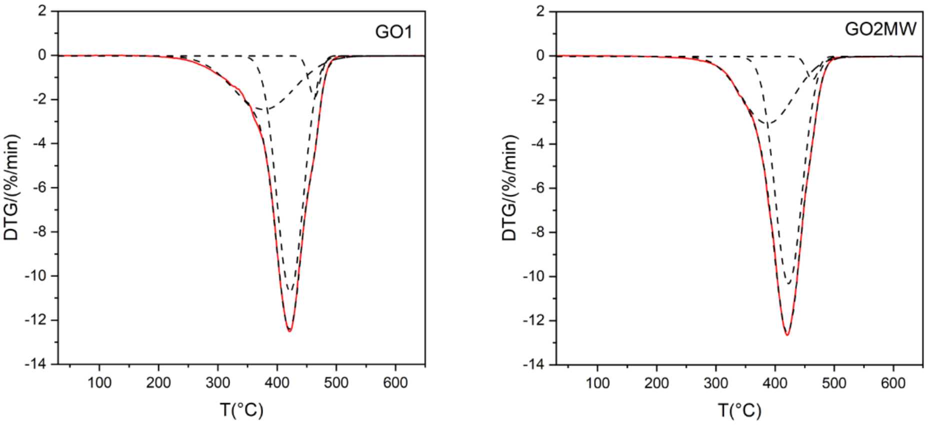

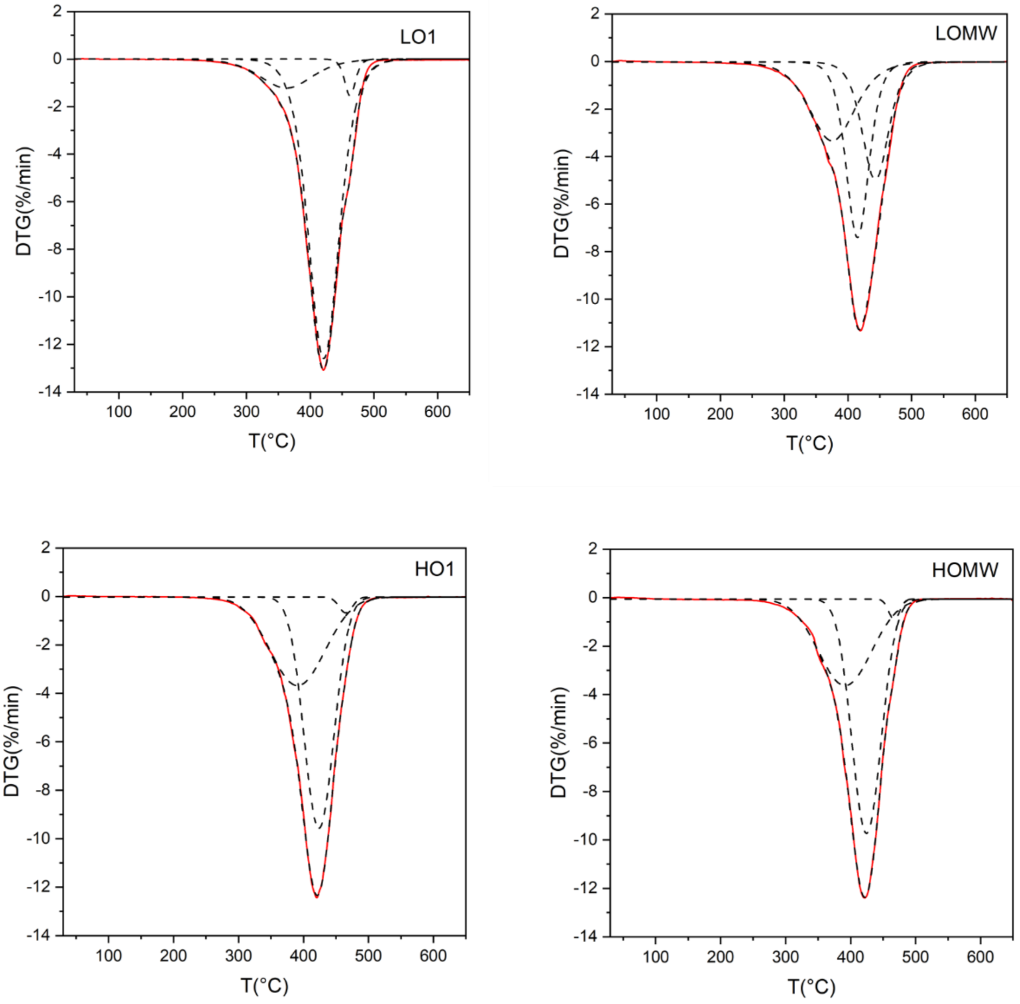

TABLE S4. Area % calculation for GO1, GO2MW, HO1, HOMW, LO1, and LOMW from the deconvolution of the DTG curves into three components. The peaks at lower temperatures are due to the decomposition of the ASA moieties: the corresponding ratios between the area of the samples obtained by microwave irradiation and those by conventional heating are 1,1, 1.0 and 2.4 for GO, HO and LO derivatives, respectively.

Sample, fitting model	Peak (°C)	Area%	r2
GO1, Gaussian	462,9	4,8	1,000
	421,9	62,5	
	377,3	32,7	

GO2MW, Gaussian	463,1<br>422.7<br>387.1	2,7<br>62.4<br>34.9	1.000
HO1, Gaussian	465,2<br>423,8<br>390,9	1,8<br>57,6<br>40,6	1.000
HOMW, Gaussian	467,0<br>424,5<br>391,5	1,9<br>58,9<br>39,2	1.000
LO1, Logistic pk	462,5<br>421,3<br>362,5	3,6<br>82,6<br>13,8	1.000
LOMW, Logistic pk	443,1<br>415,1<br>375,3	29,1<br>37,4<br>33,6	0.999
